# Multi-Layer Wear and Tool Life Calculation for Forging Applications Considering Dynamical Hardness Modeling and Nitrided Layer Degradation

**DOI:** 10.3390/ma14010104

**Published:** 2020-12-29

**Authors:** Bernd-Arno Behrens, Kai Brunotte, Hendrik Wester, Marcel Rothgänger, Felix Müller

**Affiliations:** Institute of Metal Forming and Forming Machines, Leibniz University Hannover, 30823 Garbsen, Germany; behrens@ifum.uni-hannover.de (B.-A.B.); brunotte@ifum.uni-hannover.de (K.B.); wester@ifum.uni-hannover.de (H.W.); m.rothgaenger@ifum.uni-hannover.de (M.R.)

**Keywords:** forging, wear calculation, hardness modeling, nitrided layer, Archard model, tool life

## Abstract

As one of the oldest shaping manufacturing processes, forging and especially hot forging is characterized by extreme loads on the tool. The thermal load in particular is able to cause constant changes in the hardness of the surface layer, which in turn has a decisive influence on the numerical estimation of wear. Thus, also during numerical wear, modeling hardness changes need to be taken into account. Within the scope of this paper, a new implementation of a numerical wear model is presented, which, in addition to dynamic hardness models for the base material, can also take into account the properties of a nitride wear protection layer as a function of the wear depth. After a functional representation, the new model is applied to the wear calculation of a multi-stage industrial hot forging process. The applicability of the new implementation is validated by the evaluation of the occurring hardness, wear depths and the locally associated removal of the wear protection layer. Consecutively, a tool life calculation module based on the calculated wear depth is implemented and demonstrated. In general, a good agreement of the results is achieved, making the model suitable for detailed 2D as well as large 3D Finite Element calculations.

## 1. Introduction

Due to high process temperatures, large effective stresses and extreme press kinematics, hot forging is still a production process at the limits of the technical capabilities. Due to this, the forming tools are subject to a complex load spectrum consisting of mechanical, tribological, thermal and chemical stress components [1,2]. Generally, the above-mentioned stresses occur together and, in many cases, are affecting each other. As the tool costs account for a significant share of approx. 10% of the total process costs, the research of wear protection to increase even longer tool life is of high interest [3]. A first essential step in this direction is the use of high-quality tool steels such as H10 (DIN 1.2367) or H11 (DIN 1.2343) with a process-dependent heat treatment to adjust the tool hardness [4]. The tool hardness is a significant parameter for the reduction of abrasive wear, which in turn is one of the main causes of failure of forging dies [5]. Another major contribution is made by the use of wear protection layers, such as nitrided layers, which are applied to the tool edge layer after the case hardening of the base tool steel. With these protective layers, it is possible to further increase the hardness of the surface to over 1000 HV and thus significantly extend the tool life [6,7]. Due to the high amount of influences and reactions on the tool surface layer during the forging process, it is not possible to reliably estimate the wear behavior of the tool system with conventional industrial methods such as by experience or simple rough calculations.

The use of Finite Element (FE)-supported wear calculations during process design is thus to be regarded as an essential basis for further development work in the field of wear estimation. However, most of the implemented models still rely on the original abrasive wear model developed by Archard and Holm [8,9]. In their approach, given by Equation (1), the wear *W* is calculated by the product of the normal force *F* on the surface, the sliding distance *S* and the hardness *H*. In addition, a wear factor *k* is necessary to calibrate the model on the basis of process-specific parameters, such as the material properties.

(1) $$W\;=\;k\;\cdot \;\frac{\;F\;\cdot S}{H}$$

While the parameters *F* and *S* in this model are directly derived from the respective process calculation, the tool hardness *H* plays an important role as a wear-damping or wear resistant quotient. Even if an indefinite increase in hardness is not desirable in relation to the total failure spectrum [10], this approach demonstrates that increasing the hardness of a tool contributes directly to a reduction of abrasive wear. Based on this equation, many continuing approaches exist in literature to increase the applicability of the model, for example, in modern hot forming processes. Painter et al. proposed a temperature-dependent hardness *H(T)* and three calibration constants for weighting the process variables and the tool hardness, which allows a process- and material-dependent weighting of the influencing variables on tool wear [11]. Groseclose et al. investigated a weighting of the quotient of load and hardness in relation to the sliding path by using a further coefficient [12]. Bobke developed an extended calculation approach to describe the wear of forging tools. In this approach, the tool hardness is described as a function of the cycles and is provided with an exponent [13]. This is the first time that a change in the material characteristics as a function of forging cycles is described. Behrens et al. adapted the wear model of Archard, as given in Equation (2), especially for the application in hot forging processes to consider the tempering effects [14]. By redesigning the original model in form of an incremental approach, it is possible to implement it in the context of FE-simulations. For this purpose, the wear in each calculation increment $\Delta w_{inc}$ is determined and multiplied with the incremental duration Δt. Furthermore, instead of the contact normal force, the contact normal stress $\sigma_{N\;}$ is used in conjunction with the sliding velocity $v_{rel}$. Finally, the total wear *W* for a complete production cycle is calculated by adding up all wear increments noted in Equation (3).

(2) $$\Delta w_{inc}\;=\;k\;\cdot \frac{\sigma_{N\;}\;\cdot {\;v}_{re\;l}\;\cdot \;\Delta t}{H(t,T)}$$

(3) $$W\;=\;\overset{n}{\underset{inc\;=\;1}{\sum }}\;\cdot w_{inc}$$

For the consideration of microstructure transformation processes, which have a direct influence on the tool hardness *H*, this model is able to flexibly store dynamic functions for the hardness. In the original implementation by Behrens et al., the process duration *t* and the peak temperature *T* were suggested [14].

In an analogous approach by Groseclose et al., the thermally induced microstructure transformation behavior of the tool is described with a series of tempering parameters as a function of the temperature occurring in the process. In addition, the hardness increasing influence of a nitriding protection layer in dependence of the surface layer distance is considered [15]. While the consideration of nitriding layers in wear calculations with the Archard model is highly relevant in general, the use of the surface layer distance to retrieve the corresponding tool hardness during FE-calculations is problematic. Since typical nitriding depths achievable in industrial applications reach values of approximately 0.1 mm to 0.4 mm [16,17], it is necessary to apply an even smaller element size for the discretization of the surface layer. Considering this, Groseclose could successfully use this model in a 2D calculation of an extrusion process featuring relatively simple tool geometries while maintaining the necessary small element size. However, this requirement presents a notable limitation when complex tool geometries are considered, forcing the use of a 3D calculation and making it therefore impossible to maintain economical computing times.

**Figure 1:**
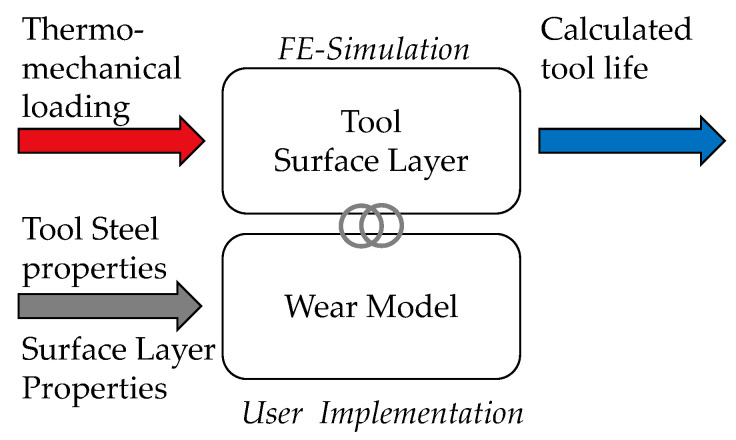
Scope of this paper: Novel approach for the wear model implementation on the tool surface layer.

To address this issue, in this work, a novel approach for the implementation of the wear model by Behrens et al. [14] is presented and discussed. The essential features of the developed approach are shown in Figure 1. To solve the problem with the near surface discretization, the wear calculation is only implemented on the outer surface layer nodes, which are also in contact with the work piece. Taking into account the thermo-mechanical tool load, the respective layer properties are referenced by using the calculated wear depth. In this way, not only different layer models can be used for the calculation of the tool life, but also the continuous removal of the top nitrided layer can be taken into account. After a detailed overview about the employed wear model, it is applied to the FE-simulation of a three stage industrial forging process and validated by an evaluation of the real tool surface concerning hardness changes and wear depth.

## 2. Materials and Methods

### 2.1. Material Modeling

**Figure 2:**
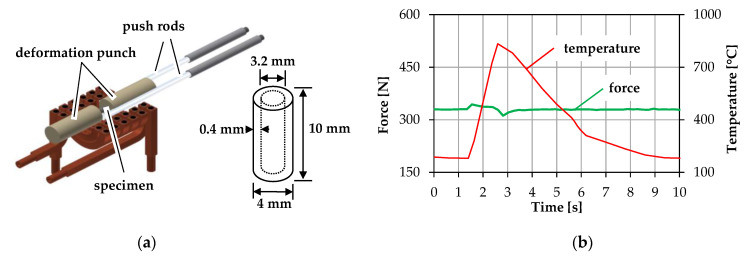
(**a**) Dilatometer DIL805D test apparatus and specimen; (**b**) Testing program to represent one forging cycle.

The surface layer of the forging die is exposed to high thermal stresses during hot forming. In previous work, it could be determined that the peak temperature in the tool edge layer can reach up to 900 °C using the common H11 (DIN 1.2343) tool steel [18]. To investigate the influence of this wide temperature range on the microstructure of common tool steels, a new process-oriented characterization method was developed, in which the tool load in terms of temperature and mechanical load can be cyclically applied to laboratory samples in a dilatometer DIL805A/D from TA Instruments [19]. Using this method, hollow samples were repeatedly heated and quenched under constant pressure in order to reproduce the thermomechanical load in a forging tool during one production cycle (Figure 2). After a defined amount of cycles, the samples were prepared for metallographical analysis in terms of hardness measurements and light microscopical images.

**Figure 3:**
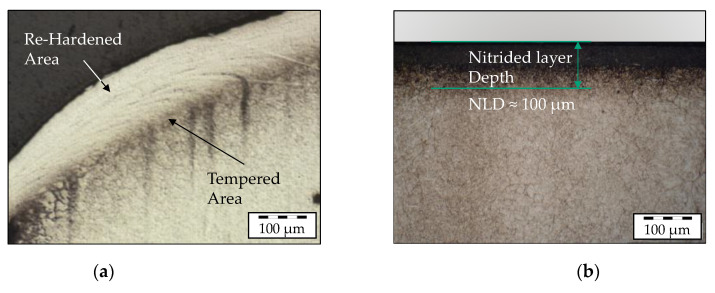
(**a**) Re-hardening and tempering effects in the tool surface layer of a forging tool; (**b**) Nitrided layer of forging tool.

Regarding the hardened base steel, this method can be used to verify the re-hardening and tempering effects, as shown in Figure 3a, with respect to the cycle-dependent hardness change. In addition to these two effects, the hardness increasing effect of a nitrided protective layer, as shown in Figure 3b, and the incremental degradation of the layer due to wear shall be considered.

**Figure 4:**
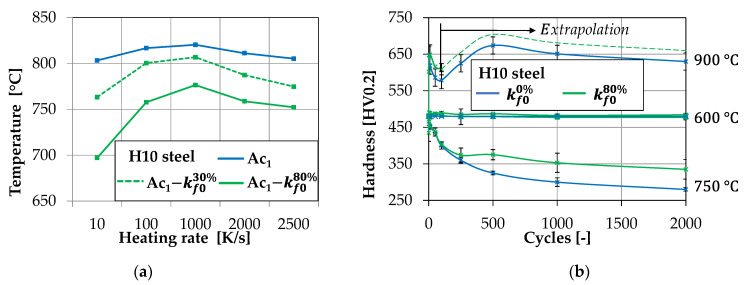
(**a**) *A_c1_* temperature of H10 steel regarding heating rate and mech. stress superposition; (**b**) Hardness evolution of H10 steel obtained by laboratory testing.

Regarding the hardness evolution, the widely used tool steel H10 (DIN 1.2367, chemical composition given in Table A1; see Appendix A) was characterized using the mentioned testing procedure in a temperature range from 600 °C up to 900 °C. All tests were performed without and with mechanical stress superposition from 30% up to 80% of the elastic stress limit *k_f0_*. The stress superposition plays an important role for the characterization of the austinization start temperature *A_c1,_* as described in Figure 4a. By increasing the mech. stress, a decrease of the *A_c1_* temperature could be observed by Malik et al., increasing the occurrence of re-hardening effects during the forging process [20]. An additional effect of the stress superposition is noticeable when performing tests around a temperature of 750 °C where tempering effects (reduction of hardness) take place. As presented in Figure 4b, the loss of hardness is thereby reduced due to a limitation of carbon diffusion by mechanical stress superposition [21].

In order to take the hardness increasing properties of a nitrided layer into account, for the first implementation, a typical nitrided layer with a thickness of 100 µm is considered. This value is derived from the reference surface layer profile of a sample shown in Figure 3b, which was taken from the validation tools (see Section 3). The reference surface profile was evaluated by using conventional Vickers hardness testing, and an average hardness of 750 HV0.2 was determined for this layer. All remaining material data of the tool steel H10 regarding, for example, the thermal or mechanical properties, were obtained from the literature [22,23].

### 2.2. Wear Modeling and Implementation

As a starting point for the wear modeling, the modified version of the Archard model by Behrens et al. is used as it offers all the flexibility needed for the implementation of the given material data presented in Section 2 [14]. For the practical demonstration of the wear model, an implementation in simufact forming 16.0 was developed using user subroutines. In general, the functions of the implementation is splitted in two main parts. The first part serves as a basic data collector placed directly during the FE-process calculation, while the second part is set up as a post-processing operation featuring a direct data transfer from the first part.

**Figure 5:**
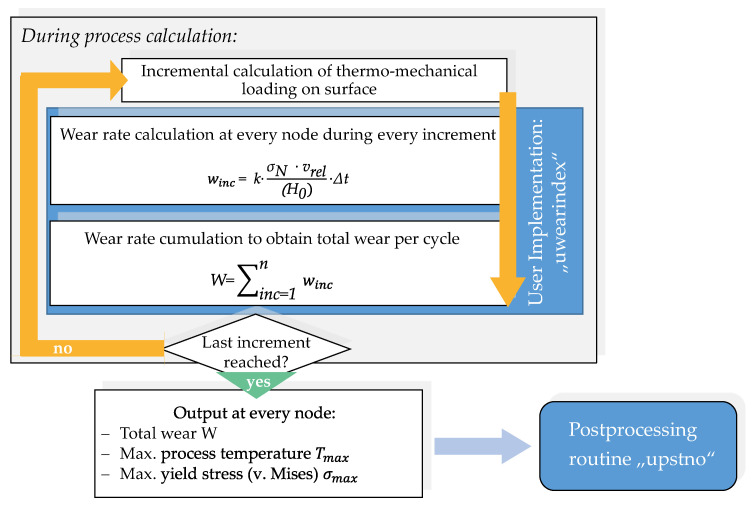
Basic wear implementation during process calculation.

To enable the wear calculation of large 3D models in an economic period, the implementation of wear modeling always requires data saving techniques to limit calculation times. To achieve this, the first part of the implementation is set up by using the UWEARINDEX user routine template provided by simufact forming, as it is only called on nodes that are currently in contact with the workpiece greatly limiting the amount of subroutine calls. As presented in Figure 5, this routine serves the purpose to pre-calculate the occurring wear during every increment and to determine the maximum process temperature and maximum yield stress at every wear-relevant node. These parameters are required for referencing the correct hardness value in the second part of the implementation. For this part of the implementation, no additional user input is required. The necessary *k* value is set to 3 × 10^−7^ based on the recommendations of Landolt and Mischler for the abrasive wear of tool steels and is not changed in the calculations of this study [24]. After the completion of the regular FE process calculation, the described results are transferred directly to the linked user routine UPSTNO. This subroutine type is mandatory for the display of user variables in the user interface and is used for further processing of wear and process data.

**Figure 6:**
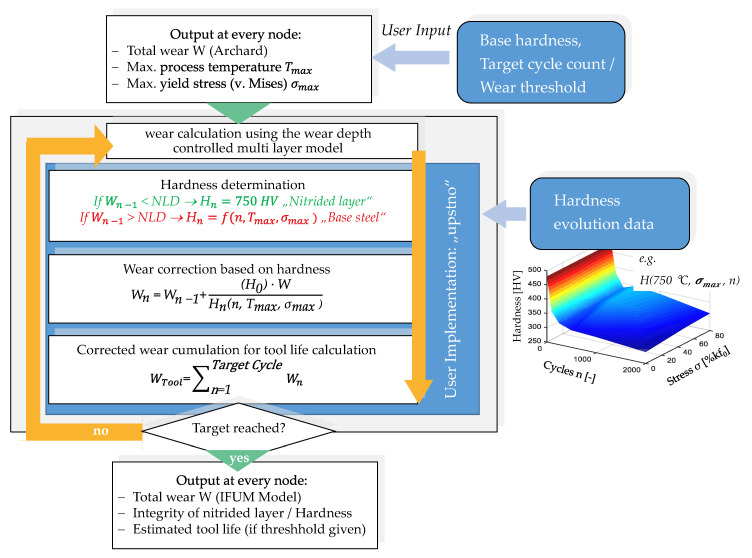
Hardness and wear model implementation during post-processing.

The method of the following calculation of hardness change and the cyclic-dependent update of the pre-calculated wear of the initial cycle is displayed in Figure 6. The main purpose of this routine is the extrapolation of the occurring wear *W* while constantly reevaluating the hardness. This task is carried out by evaluating, at first, the already calculated wear depth *W_n−1_* to determine the integrity of the nitrided layer. If the named value exceeds the nitrided layer depth (NLD), the layer is marked as degraded and the hardness evolution data of the base steel is referenced. If not, the hardness at this node is set to 750 HV, representing the nitride layer hardness.

It has to be pointed out, that even if the hardness of the nitride layer is used for the wear calibration, the base material is still subject to hardness evolution since the comparatively thin nitrided layer (0.1 mm in this case) does not noticeably reduce the thermal load on the base material.

The hardness calculation of the base material is achieved by importing the hardness evolution data, shown in Figure 4b, formatted as tabular data at the given measuring points into the user routine. By calling the maximum values for the process temperature and the yield stress at each node, a hardness value is calculated using a fast to perform, linear interpolation between the given data points. After the hardness calculation, the further method depends on the user input regarding the calculation target. For model validation purposes, the routine can be used by providing a discrete target cycle count to display the local amount of wear at a set cycle number. This method is mainly demonstrated in Section 3. Another termination criterion can be set up by defining a discrete maximum wear depth at which the tool is considered as worn out. In this case, the main result of the routine is given by an expectable tool life value. However, to ensure a reliable result, which is not affected by numerical instabilities like overshooting nodes, which can never be completely avoided during large 3D calculation, an additional stability criterion has to be defined. This is achieved by setting up a minimum node threshold number depending on the amount of total simulation nodes. By doing this, the expected tool life is only achieved if a defined number of nodes exceeds the defined limit for the wear depth during the wear extrapolation.

Since for each process cycle, a calculation loop is performed at each node as shown in Figure 6, the calculation of the lifetime of forging tools, which in reality can reach typical lifetimes of around 10,000 parts [25], quickly leads to a significant increase of the calculation time. For this reason, a cycle time-scaling, which is comparable to known techniques like mass- and time-scaling [26], is implemented. Since it can be assumed that the cycle-dependent hardness evolution does not change significantly in a consideration interval of ten cycles, the cycle step size can be increased by a factor of 10 and the wear can be corrected accordingly, which leads to a significant reduction of the calculation effort.

### 2.3. Industrial Validation Process

**Figure 7:**
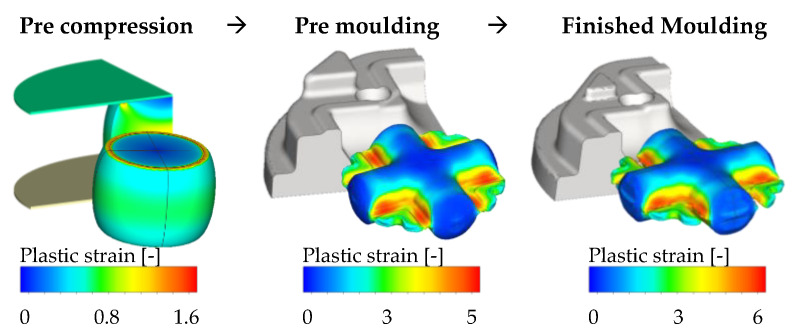
Three stage crosslink forming process at Hammerwerk Fridingen GmbH.

To demonstrate the capabilities and features of the new wear implementation, a full process calculation of a three stage crosslink forming process performed at Hammerwerk Fridingen GmbH is built up (see Figure 7). The full forming process consists of a pre-compression phase where the raw material is formed into a bulged cylinder. After that, the cylinder is premolded to achieve most of the needed material flow. During the finished molding, the preformed part is brought into near net shape by engraving the final geometry details under high mechanical load. The raw parts consist of cylinders with a diameter of 75 mm and a height of 150 mm made of AISI 1045 (DIN C45, chemical composition; see Table A3 in Appendix A). The resulting semi-finished product mass amounts to approx. 5 kg. The final parts are used in the automotive industry as part of a truck drive train. The forging operation, in general, is classified as hot forging with burr. The forging tools for all forming stages are made of H10 tool steel and case hardened to a starting hardness of 480 HV. After the case hardening, a stress relief annealing was performed to eliminate the influence of residual stresses before the start of the forming operation.

All three forming stages are performed directly, one after the other, so that the raw part is heated to 1200 °C only once before precompression. This procedure is represented in the FE calculations by transferring the calculated temperature fields of the work piece after each forming step to the next. The core temperature of the tools reaches a stationary value of approx. 200 °C during the industrial series production. Since the premolding stage is subject to high thermal and the finished molding subject to high mechanical loads, the wear of these two stages is evaluated in the following Results section. All simulation models are build up as ¼ cuts of the original geometry using the respective symmetry planes to save calculation time. All parts are discretized using tetrahedron elements. The tool material properties are marked as deformable parts to allow for a more precise calculation of the resulting contact stresses. Friction is set up for all steps using a friction factor of *m* = 0.4 for the shear friction model. The kinematics of the crank press are derived from the industry application. The premolding is evaluated at a cycle count of 17,700 while the finished molding is evaluated at 5400 cycles due to the tool conditions provided by the industry partner for validation purposes.

## 3. Results

### 3.1. Tool Hardness Calculation

**Figure 8:**
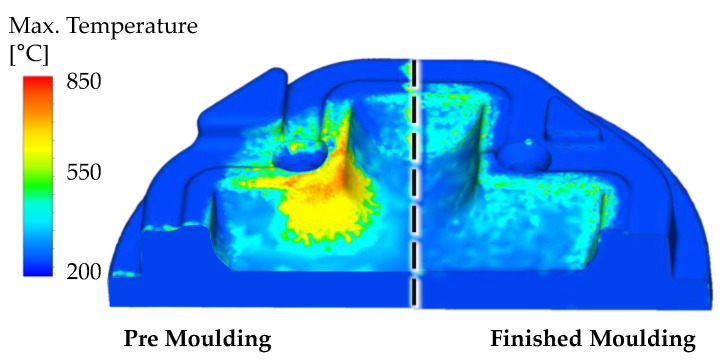
Maximum process temperature of the pre- and finished molding stage.

**Figure 9:**
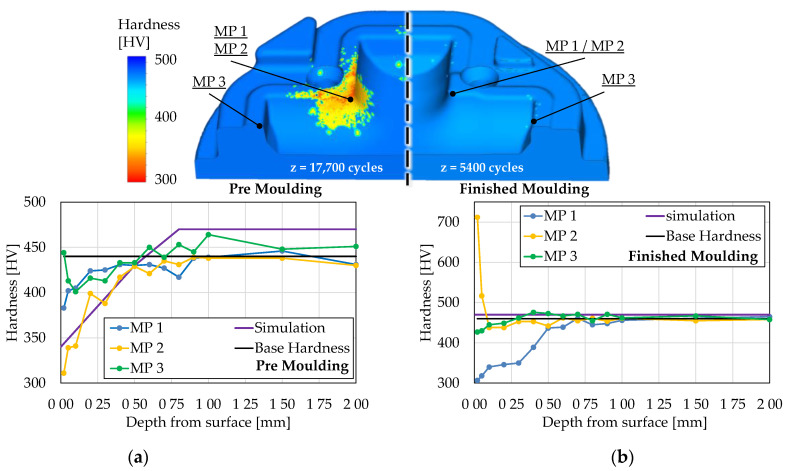
(**a**) Hardness calculation of the premolding and (**b**) finished molding stage compared to hardness measurements of the real tools.

As described during the material modeling (Section 2.1), the main parameter for the hardness evolution of the base material (H10) is given by the maximum process temperature. This value is determined by the first part of the user routine and presented in Figure 8. During premolding, the central corner of the tool is dominantly thermally loaded and reaches temperatures of approx. 800 °C. Compared to this, the loading on the finished molding stage is significantly lower, reaching temperatures of approx. 500 °C. Based on the maximal temperatures and the mechanical loadings, the premolding is subject to substantial tempering effects leading to a decrease of hardness pictured in Figure 9a. As the austenitization start temperature *A_c1_* (above 800 °C) is not reached, no re-hardening effects are expected. The hardness measurements at three relevant measuring points on the real industrial tools show a good agreement with the calculated hardness data at the given cycle count. Measuring point 1 and 2 (MP 1 and MP 2) are obtained at the same location but on different (opposing) quarters of the die. Therefore, there is only one simulation data line for comparison. A minor exception is found at measuring point 3 (MP 3), where a slight increase in hardness is noticeable. However, images of the microstructure (see chapter 3.2) could clarify that at this location, residues of the nitrided layer are still prominent, which are not considered during the visualization of the hardness calculation of the base material.

For the finished molding, similar results could be observed (Figure 9b). Since the maximum temperature does not exceed the tempering start temperature of 600 °C, no changes in hardness could be observed. The hardness measurements show a good agreement at MP 3. MP 2 presents an analogous exception where the nitrided layer is still intact, leading to hardness of over 700 HV. The tempering effects of MP 1 can be reasoned with multiple reasons, e.g., process alteration and, therefore, not explainable in the scope of this study.

### 3.2. Wear Calculation and Nitrided Layer Integrity

**Figure 10:**
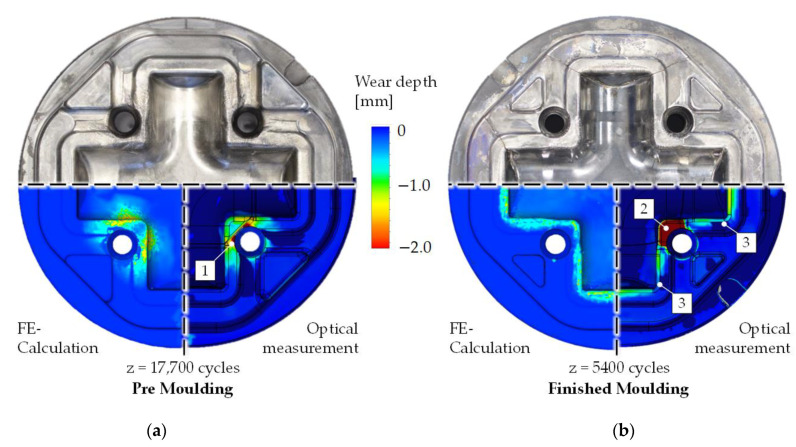
(**a**) Calibrated wear calculation of the premolding and (**b**) finished molding stage compared to optical measurements.

The next model validation is given by the results of the final wear calculation shown in Figure 10. In general, a good agreement of the wear localization is found for both forming stages. The absolute amount of wear is validated by two observation methods. At first, the worn tools were 3D scanned using the optical camera system ATOS by GOM, producing a Computer-Aided-Design (CAD) model of the worn surface. After the measurements, the output is merged with the original CAD design of the tools to highlight the worn out areas and calculate the absolute wear depth. In total, three dies per forming stage were analyzed using this technique. These results are directly comparable to the calculated wear depth of this study.

Based on the results of the wear analysis, three findings (highlighted numbers in Figure 10) concerning the comparison of the calculation are addressed:

1. The measurements of the real tools are showing an increased amount of wear close to the contour next to ejector hole. To reduce the complexity of the calculation model, these holes were covered with analytical rigid parts leading to a minor deviation of the part material flow. Consequently, the calculated wear is underestimated at this location.
2. By closer inspection of the finished molding, a high, homogenous amount of wear is found at the central corner of the mold using the optical measurements. An analysis of the calculated contact pressure and the sliding velocity at this region shows that the die is exposed to high mechanical pressure, but also that there is almost no material flow. It must therefore be assumed that the measured contour deviation cannot be explained by wear, but by deformation of the die.
3. The corners of die are subject to a low amount of wear following the calculation results. The optical measurements are unclear in this region. While one corner is showing a comparable amount of wear, the other corner shows almost no wear. This finding could be explained with deviations of the insertion position during the industrial forming which are not displayable by the FE-calculation.

**Figure 11:**
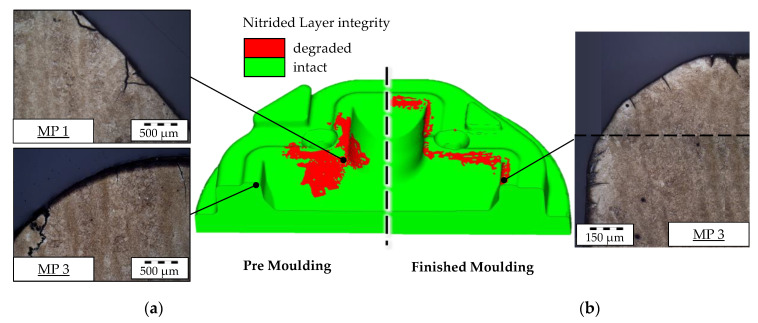
(**a**) Nitrided layer integrity calculation of the premolding and (**b**) finished molding stage compared to microscopic images of the real tool.

Another validation of the calculated absolute wear depth is given by the evaluation of the integrity of the nitrided layer as this can be clearly determined by metallographic images of the surface layer microstructure. As explained during the implementation of the model, the nitrided layer is numerically considered as degraded if a wear depth of over 0.1 mm is reached. Figure 11 shows the local integrity results. This calculation shows a good correlation to the taken microscopic images, while at MP 1 of the premolding, the nitride layer is both degraded in the calculation and the image. At MP 3, the layer shows signs of wear but is still considerable as intact in agreement to the calculation. Following this observation at MP 3 of the finished molding, a stitched image of the tool surface layer was processed showing a degradation of the layer at the outer radius, which again agrees to the numerical simulation.

Due to these two validation methods, the calculated absolute wear values are considered as valid for an additional evaluation of the expected tool life in the following Section.

### 3.3. Tool Life Calculation

**Figure 12:**
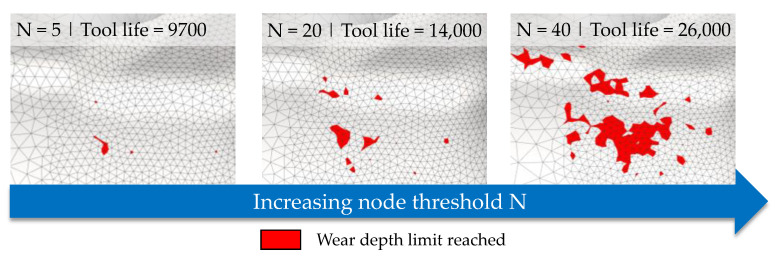
Affected tool surface area regarding the defined node threshold N.

For the calculation of tool life, two main user inputs are required. First, the acceptable wear depth for determining the worn out status of the tool is set to 2 mm as a statistically derived value from three individual dies from each stage. To investigate the influence of the implemented node threshold *N* on the tool life, a parameter study was carried out using the process simulation of the finished molding, as this process is easier to comprehend since no hardness evolution occurs. The general influence of the node threshold value *N* on the calculation results is pictured in Figure 12, showing a magnification of the central tool corner. If a low number for the threshold is chosen, the affected surface area reaching the defined wear is also low, considering the element size concerning the real tool surface. By increasing the node threshold, the affected surface area also increases. At this point, an optimization problem arises to find a threshold number leading to valid tool life counts. The challenge is to select the threshold value in such a way that the influence of overshooting nodes is compensated for, but that no more nodes need to be activated than are actually located in the wear-critical area.

**Figure 13:**
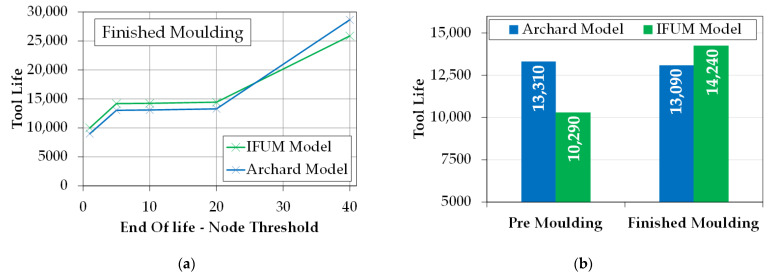
(**a**) Influence of the end of life determining node threshold on the tool life; (**b**) Expected tool life of both forming stages with model comparison to the Archard model (*H = 480; HV = const.), Node threshold = 20, acceptable wear depth = 2 mm.*

Consecutively, a parameter study is carried out by varying the node threshold in the range from 1 to 40 to calculate the tool life using the new model of the Institute of Metal Forming and Machines (IFUM) and the classical Archard model (constant hardness of *H =* 480 HV) and compared in Figure 13a. The results show that for this process, an optimal node threshold can be found between 5 and 20 nodes. If a value below this range is chosen, the impact of instabilities such as overshooting nodes greatly increases. If a value above is chosen, the node count required for reaching the threshold exceeds the number of nodes in the wear prominent regions of the die surface leading to an unrealistic jump of the tool life. Due to these findings, in Figure 13b, the tool life for both stages is compared using the IFUM and the Archard model with a node threshold of 20 and an acceptable wear depth of 2 mm.

Using these values, the tool life of the finished molding calculated with the IFUM model is nearly 10% longer as using the Archard model. Since no hardness evolution takes place during this process, the only difference between the material modeling of both models is the consideration of the nitride layer. Keeping in mind that after this layer is worn out, both models use the same hardness leading to the static increase of tool life using the IFUM model. If, on the other hand, the premolding stage is considered, in which significant hardness evolution effects occur, the tool life calculated with the IFUM model is about 25% shorter than using the Archard model. This is explained by the fact that the Archard model maintains a constant hardness of 480 HV over the full tool lifetime, while the hardness using the IFUM model drops in a relatively short time (after about 2000 cycles) to approx. 300 HV. This leads to an increase of wear of around 33% due to strong tempering effects resulting from high thermal loadings.

## 4. Discussion

### 4.1. Hardness Calculation

**Figure 14:**
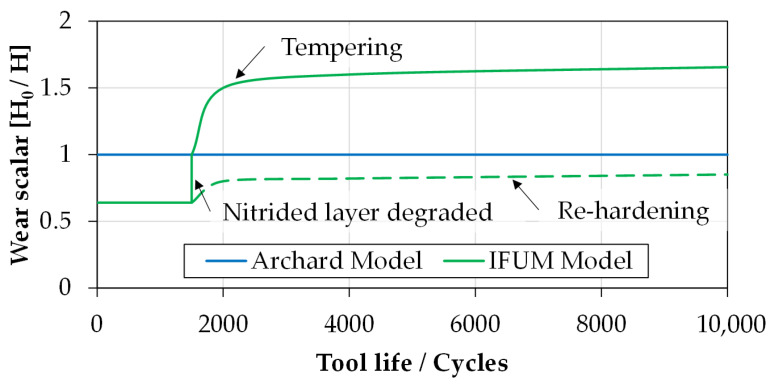
Wear scalar for calibrating the wear using the IFUM model compared to the Archard model.

As explained in Section 3.3., the results of calculated tool life are strongly depended on the occurring types of hardness changes in the surface area of the tools. While for the classic Archard model, the occurring wear is extrapolated linearly using a constant hardness *H*_0_, the wear using the IFUM model is re-evaluated by the wear scalar *H_0_/H(n, T, σ_N_)* during each life cycle. The main differences of this implementation are considered in Figure 14. After the nitrided layer is degraded, the IFUM model uses a dynamic hardness function that either considers tempering or re-hardening effects. Regarding each case, the wear scalar either assumes values above 1 if the hardness according to the IFUM model is reduced, or assumes a value below 1 if the hardness is increased, compared to the base hardness *H*_0_. Since the peak temperatures of both considered processes are below 800 °C, no re-hardening occurred.

Considering the results of the hardness calculation presented in Figure 9, the calculated hardness depth profile for the premolding die matches the experimental values at measuring point 2 (MP 2) with a maximum deviation of approx. 30 HV. The hardness measurements were carried out using the Vickers testing method with a testing load of ≈ 0.1 N (corresponds to HV0.1). The testing load was chosen to ensure a small indent size of approx. 24 µm at a hardness of 400 HV0.1. With this method, it is possible to record a valid hardness value in a depth of 100 µm from the tool surface. This measuring position is of high interest to be able to map the actual wear-relevant surface hardness. However, in accordance with the test standard DIN EN ISO 6507, a measurement error of 8–10% must be accepted when using the named test load in a hardness range of 300 HV to 400 HV [27]. Keeping this in mind, the calculated hardness is in good agreement to the measurements at MP 2. When comparing the calculation to MP 1, a deviation of approx. 50 HV is noticeable. At this location, a process deviation must be assumed during the experiment, which cannot be represented with the idealized FE-calculation. Nevertheless, a good mapping quality can also be assumed for this measuring point with the implemented model, since the occurring hardness deviation of 15–20% is still within the permissible deviation from the DIN EN standard [27].

### 4.2. Wear Calculation

As described in the introduction, the wear calculation according to Archard or IFUM approach is based on an evaluation of contact pressure, sliding velocity and hardness. Contact pressure and sliding velocity are calculated directly by the FE solver and can therefore not be validated directly from outside. Since these two variables generally determine the wear locations, the condition of the nitriding layers was considered as part of the study. Light microscopy images of the surface layer microstructure showed that the calculated wear locations basically correspond to the abrasion of the nitrided layers. The hardness changes, on the other hand, are calculated with the described IFUM model and determine directly the absolute amount of abrasive wear. Since there is a linear relationship between all variables of the wear model, it can be assumed, in the context of the study, that the error in the calculated wear primarily corresponds to the stated error in the hardness modeling of approximately 10%.

### 4.3. Tool Life Estimation

**Figure 15:**
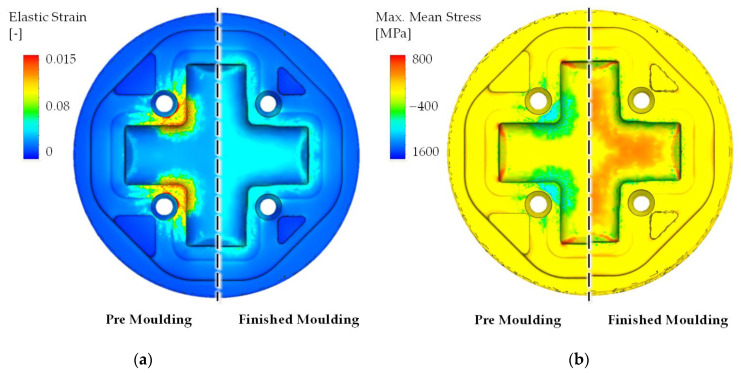
(**a**) Elastic strain and (**b**) Maximum mean stress distribution of both forming stages.

While the calculated values for the tool life are in a plausible relation to each other, the feature still has to be considered as experimental. As a first approach, in this study, the calibration parameters for this calculation (node threshold, cycle-time-scaling factor) where handpicked based on experience or carrying out a parameter study and are, therefore, applicable only for the considered forming process. To further increase the usability of this calculation method, the evaluation of the area assigned to each surface node could be considered. By adding this parameter to the acceptable wear depth and the node threshold, a more easily comprehensible wear volume can be calculated to determine the end of tool life. Following the argumentation of Jafors et al. [25], the tool life of a single die must always be considered as a single data point which is subject to strong fluctuations in the industrial manufacturing process [28]. Against this background, the calculated tool life represents a statistical mean value which can only be compared to a statistical evaluation of the lifetime over a longer period of time. Therefore, for this study, the tool life is calculated using the new IFUM model and compared to the calculation results using the industrially established Archard model. Since it is not possible to determine the exact cause of failure of the dies provided for the validation of the wear depth afterwards, a direct comparison of the calculated tool life with the individual dies provided is not applicable. In addition to the considered abrasive wear, cracking must be considered as a significant cause of failure limiting the tool life of the die. Following the fatigue/crack models of Basquin [29] or Goodman [30], the main influencing factors for the ignition of cracks can be found in cyclic strain and mean stress loading. These parameters are displayed in Figure 15 for the two processes under consideration. There, the occurrence of significant tensile stresses in conjunction with an elastic strain component is particularly noticeable at the end of the respective cross arms, which can lead to the formation of cracks in the course of a cyclic load. Since the FE-model was calculated thermo-mechanically coupled, the resulting stresses are directly influenced by mechanical loads as well as strains by thermal gradients. The areas where abrasive wear occurs, on the other hand, are characterized by significant compressive stresses. Due to these findings, a preliminary failure of the tools before reaching the set wear limit is possible, but is not taken into account by the implemented wear modeling at this point in time.

## 5. Conclusions

In the present study, a new wear model based on surface nodes was implemented. By means of this adaptation and the extensive consideration of properties of the tool edge layer, such as dynamic hardness changes and the wear depth controlled consideration of a nitrided layer, a high degree of realism is targeted. Furthermore, with this implementation, it is possible to consider large 3D FE calculation models.

As part of the validation, a two-stage industrial process of Hammerwerk Fridingen GmbH was numerically investigated with regard to the hardness changes in the tool surface layer, the occurring wear and the tool life. Starting from an initial hardness of 480 HV, the implemented model successfully predicted a tempering of the tool surface layer, where the real measured hardness depth profile could be predicted with a deviation of less than 30 HV. Overall, the hardness in the surface layer of the preform tools decreased to approx. 300 HV due to the thermal load. No hardness changes were calculated during the finishing die stage because the necessary tempering start temperature of 600 °C was not reached on the die surface. With regard to the calculated wear, good prediction accuracies were achieved in the localization of the wear locations. The wear depth determined in the experiment of just under 2 mm in the preform stage (after 17,700 cycles) and approx. 1.5 mm in the final forming stage (after 5400 cycles) could be predicted with the implemented wear model. To predict the tool life, a criterion was implemented based on the calculated wear depth. Additionally, a node threshold number is set in place to increase the numerical stability. Using the tool life model, the differences between the new IFUM wear model and the classic model by Archard was demonstrated. It was shown that a reduction in tool life of approx. 30% can be expected due to the hardness changes resulting from the thermomechanical load. In connection with the described calculation error of the hardness and wear model of approx. 10%, it can thus be summarized that the implementation of the multi-layer wear model with dynamic hardness consideration leads to an increase in the tool life prediction quality when compared to the classic Archard model.

## 6. Future Work

Depending on the available computing capacity, the model can easily be adapted to carry out the IFUM wear calibration already during the process calculation (before the last increment). This makes it possible to determine the calibrated wear value as a function of the increment time or the press stroke at the cost of a longer calculation time. In this way, it is possible to detect additional potentials for further process optimization.

Whereas for the material characterization of the base material within the scope of this study, a very detailed modeling could be referenced, a single static hardness value in connection with a constant layer thickness was defined as a first approach for the properties of the nitrided protection layer. As the properties of this layer in terms of hardness during the adjustment process are essentially determined by the set temperatures and treatment times [17,28], it can be assumed that the properties of this layer also undergo an evolution during the ongoing process. For this reason, a characterization analogous to the one described by Müller et al. [19] is already in progress in order to implement the results in the next iteration into the model described here. The necessary validation tests will be carried out using cyclic forging tests under laboratory conditions. This will be adjusted in order to achieve temperatures on the tool surface in a wide range between 600 °C and 900 °C by variation of press kinematics. This ensures that all implemented temperature-controlled material effects are considered to enable a full validation of the wear modeling.

Considering the findings regarding the mean stresses and the risk of preliminary tool failure before reaching the abrasive wear limit due to cracking, it is planned to combine the IFUM abrasive wear model with a stress- and strain-based fatigue model using a material parameter set for the description of H10 tool steel obtained by Behrens et al. [31]. By a continuous, parallel check of the abrasive wear criterion and the failure cycle number calculated by a stress-based failure criterion for cyclic tool damage, the tool life can be determined when one of the models predict a critical value.

## Data Availability

The data presented in this study are available on request from the corresponding author. The data are not publicly available due to industrial confidentiality.

## Appendix A

**Table A1:** Chemical composition of H10 tool steel (DIN 1.2367) in mass % [22].

C	Cr	Mo	V	Si
0.37	5.0	3.0	0.6	0.3

**Table A2:** Chemical composition of H11 tool steel (DIN 1.2343) in mass % [22].

C	Cr	Mo	V	Si
0.37	5.0	1.25	0.4	1.0

**Table A3:** Chemical composition of AISI 1045 (DIN C45) in mass % (provided by the supplier).

C	Mn	P	S
0.45	0.75	Max. 0.04	Max. 0.05
